# Supplementary material for: Association study based on topological constraints of protein–protein interaction networks
Source: Sci Rep. 2020 Jul 1;10:10797. doi: 10.1038/s41598-020-67875-w (PMC7329836; doi:10.1038/s41598-020-67875-w)
Supplement: Supplementary file 1 — Supplementary file1 (DOCX 14437 kb) [file 41598_2020_67875_MOESM1_ESM.docx]

Supplementary Information for

**Association Study based on Topological Constraints of Protein-Protein Interaction Networks**

Hao-Bo Guo^1,2^*, Hong Qin^1,2,3*^

^1^Department of Computer Science and Engineering

^2^SimCenter

^3^Department of Biology, Geology and Environmental Science

The University of Tennessee at Chattanooga

* Correspondence: haobo-guo03@utc.edu and hong-qin@utc.edu

*A list of the Supplementary Information*

**Table S1**. List of 50 hallmark protein sets from MSigDB

**Table S2**. Summary of 19 human disorders used in present work

**Table S3**. Summary of the 50 hallmark sets from MSigDB

**Figure S1** Normality test of the interaction numbers from null models and comparison of the Z-score and modified Z’-score heatmaps of the hallmark-hallmark interactions.

**Figure S2**. Heatmaps based on the p-values and q-values for evaluating the associations among the 50 hallmark gene sets.

**Figure S3** Examples indicate that overrepresentation is not equivalent to enriched interactions, and that the interaction numbers from the PIN may be suppressed compared to the null network models.

**Figure S4**. Interaction Z-score heatmaps of randomly constructed gene sets

**Figure S5**. Comparisons of NetPAS, DAVID and WebGestalt using synthetic gene sets.

**Table S1. List of 50 hallmark protein sets from MSigDB**

1. HALLMARK_ADIPOGENESIS,

2. HALLMARK_ALLOGRAFT_REJECTION,

3. HALLMARK_ANDROGEN_RESPONSE,

4. HALLMARK_ANGIOGENESIS,

5. HALLMARK_APICAL_JUNCTION,

6. HALLMARK_APICAL_SURFACE,

7. HALLMARK_APOPTOSIS,

8. HALLMARK_BILE_ACID_METABOLISM,

9. HALLMARK_CHOLESTEROL_HOMEOSTASIS,

10. HALLMARK_COAGULATION,

11. HALLMARK_COMPLEMENT,

12. HALLMARK_DNA_REPAIR,

13. HALLMARK_E2F_TARGETS,

14. HALLMARK_EPITHELIAL_MESENCHYMAL_TRANSITION,

15. HALLMARK_ESTROGEN_RESPONSE_EARLY,

16. HALLMARK_ESTROGEN_RESPONSE_LATE,

17. HALLMARK_FATTY_ACID_METABOLISM,

18. HALLMARK_G2M_CHECKPOINT,

19. HALLMARK_GLYCOLYSIS,

20. HALLMARK_HEDGEHOG_SIGNALING,

21. HALLMARK_HEME_METABOLISM,

22. HALLMARK_HYPOXIA,

23. HALLMARK_IL2_STAT5_SIGNALING,

24. HALLMARK_IL6_JAK_STAT3_SIGNALING,

25. HALLMARK_INFLAMMATORY_RESPONSE,

26. HALLMARK_INTERFERON_ALPHA_RESPONSE,

27. HALLMARK_INTERFERON_GAMMA_RESPONSE,

28. HALLMARK_KRAS_SIGNALING_DN,

29. HALLMARK_KRAS_SIGNALING_UP,

30. HALLMARK_MITOTIC_SPINDLE,

31. HALLMARK_MTORC1_SIGNALING,

32. HALLMARK_MYC_TARGETS_V1,

33. HALLMARK_MYC_TARGETS_V2,

34. HALLMARK_MYOGENESIS,

35. HALLMARK_NOTCH_SIGNALING,

36. HALLMARK_OXIDATIVE_PHOSPHORYLATION,

37. HALLMARK_P53_PATHWAY,

38. HALLMARK_PANCREAS_BETA_CELLS,

39. HALLMARK_PEROXISOME,

40. HALLMARK_PI3K_AKT_MTOR_SIGNALING,

41. HALLMARK_PROTEIN_SECRETION,

42. HALLMARK_REACTIVE_OXIGEN_SPECIES_PATHWAY,

43. HALLMARK_SPERMATOGENESIS,

44. HALLMARK_TGF_BETA_SIGNALING,

45. HALLMARK_TNFA_SIGNALING_VIA_NFKB,

46. HALLMARK_UNFOLDED_PROTEIN_RESPONSE,

47. HALLMARK_UV_RESPONSE_DN,

48. HALLMARK_UV_RESPONSE_UP,

49. HALLMARK_WNT_BETA_CATENIN_SIGNALING,

50. HALLMARK_XENOBIOTIC_METABOLISM.

**Table S2**. Summary of 19 human disorders used in present work

| MIM ID | Name | Name in Figure | Gene Number |
| --- | --- | --- | --- |
| 114480 | Breast Cancer | Breast | 22 |
| 114500 | Colorectal Cancer | Colorectal | 26 |
| 211980 | Lung Cancer | Lung | 14 |
| 167000 | Ovarian Cancer | Ovarian | 6 |
| 114550 | Hepatocellular Carcinoma | HCC | 9 |
| 133239 | Esophageal Cancer | Esophageal | 5 |
| 613659 | Gastric Cancer | Gastric | 8 |
| 176807 | Prostate Cancer | Prostate | 9 |
| 144700 | Renal Cell Carcinoma | RCC | 7 |
| 601626 | Acute Myeloid Leukemia | AML | 20 |
| 607174 | Meningioma | Meningioma | 6 |
| 171300 | Pheochromocytoma | Pheoch | 8 |
| 605027 | Non-Hodgkin Lymphoma | NHL | 5 |
| 104300 | Alzheimer | Alzheimer | 6 |
| 608516 | Major Depression Disorder | MDD | 5 |
| 181500 | Schizophrenia | Schizophrenia | 21 |
| 601665 | Obesity | Obesity | 11 |
| 168600 | Parkinson | Parkinson | 7 |
| 125853 | Diabetes Mellitus, Type II | Diabete2 | 30 |

**Table S3**. Summary of the 50 hallmark sets from MSigDB

| **Set** | **V** | **E** | **Iso** | **D_max_** | **CC** | **Clust** | **Cliq** | **BP_max_** | **CC_max_** | **MF_max_** |
| --- | --- | --- | --- | --- | --- | --- | --- | --- | --- | --- |
| 1 | 200 | 445 | 29 | 159 | 0.123 | 171 | 10 | 18.434 | 26.675 | 18.010 |
| 2 | 200 | 616 | 26 | 40 | 0.398 | 166 | 11 | 55.890 | 50.323 | 46.862 |
| 3 | 101 | 55 | 52 | 10 | 0.132 | 39 | 3 | 13.983 | 9.221 | 13.150 |
| 4 | 36 | 25 | 16 | 6 | 0.224 | 20 | 3 | 32.793 | 23.620 | 24.619 |
| 5 | 200 | 570 | 42 | 32 | 0.299 | 150 | 7 | 36.113 | 40.408 | 38.653 |
| 6 | 44 | 15 | 27 | 5 | 0.111 | 13 | 3 | 14.174 | 16.512 | 23.880 |
| 7 | 161 | 467 | 23 | 42 | 0.215 | 138 | 7 | 27.164 | 19.898 | 28.141 |
| 8 | 112 | 89 | 53 | 10 | 0.434 | 53 | 6 | 40.115 | 43.054 | 52.303 |
| 9 | 74 | 46 | 42 | 10 | 0.379 | 26 | 5 | 11.587 | 11.525 | 10.507 |
| 10 | 138 | 260 | 36 | 28 | 0.203 | 98 | 5 | 47.730 | 45.398 | 53.650 |
| 11 | 200 | 356 | 55 | 27 | 0.242 | 143 | 7 | 40.962 | 43.591 | 36.086 |
| 12 | 150 | 1276 | 37 | 63 | 0.639 | 113 | 31 | 83.368 | 58.951 | 48.788 |
| 13 | 200 | 1713 | 9 | 73 | 0.375 | 191 | 22 | 47.567 | 45.133 | 28.487 |
| 14 | 200 | 503 | 45 | 31 | 0.221 | 150 | 7 | 66.655 | 70.180 | 72.791 |
| 15 | 200 | 134 | 110 | 31 | 0.109 | 74 | 4 | 23.846 | 13.435 | 21.414 |
| 16 | 200 | 106 | 112 | 11 | 0.197 | 69 | 5 | 16.162 | 20.635 | 16.505 |
| 17 | 158 | 337 | 62 | 38 | 0.365 | 94 | 11 | 22.927 | 32.894 | 26.219 |
| 18 | 200 | 1597 | 14 | 80 | 0.332 | 184 | 22 | 41.826 | 44.836 | 23.088 |
| 19 | 200 | 605 | 75 | 39 | 0.529 | 125 | 14 | 51.240 | 29.767 | 40.619 |
| 20 | 36 | 21 | 15 | 6 | 0.375 | 13 | 4 | 32.110 | 28.148 | 28.148 |
| 21 | 200 | 111 | 97 | 10 | 0.203 | 74 | 4 | 15.614 | 12.122 | 13.595 |
| 22 | 200 | 435 | 64 | 42 | 0.414 | 132 | 12 | 35.609 | 22.241 | 26.900 |
| 23 | 200 | 145 | 83 | 34 | 0.085 | 98 | 4 | 18.847 | 19.822 | 13.461 |
| 24 | 87 | 206 | 12 | 23 | 0.348 | 64 | 8 | 58.317 | 46.169 | 60.263 |
| 25 | 200 | 410 | 66 | 29 | 0.717 | 123 | 18 | 157.204 | 96.487 | 107.292 |
| 26 | 97 | 65 | 51 | 10 | 0.325 | 38 | 5 | 26.563 | 20.985 | 19.913 |
| 27 | 200 | 371 | 62 | 22 | 0.334 | 134 | 8 | 47.691 | 26.453 | 49.197 |
| 28 | 200 | 93 | 126 | 9 | 0.852 | 14 | 9 | 70.259 | 40.905 | 52.186 |
| 29 | 200 | 83 | 127 | 8 | 0.528 | 15 | 5 | 19.441 | 22.030 | 17.106 |
| 30 | 200 | 1228 | 13 | 60 | 0.467 | 183 | 25 | 66.593 | 66.347 | 40.223 |
| 31 | 200 | 903 | 43 | 56 | 0.416 | 155 | 15 | 21.195 | 31.196 | 21.145 |
| 32 | 200 | 3018 | 7 | 101 | 0.448 | 193 | 31 | 32.985 | 30.177 | 23.744 |
| 33 | 58 | 181 | 15 | 24 | 0.551 | 43 | 10 | 40.305 | 36.847 | 22.212 |
| 34 | 200 | 628 | 48 | 38 | 0.507 | 152 | 16 | 73.619 | 56.463 | 59.723 |
| 35 | 32 | 68 | 3 | 18 | 0.318 | 29 | 5 | 28.680 | 22.770 | 29.708 |
| 36 | 200 | 1380 | 24 | 68 | 0.601 | 176 | 28 | 158.174 | 144.857 | 143.608 |
| 37 | 200 | 342 | 57 | 41 | 0.181 | 141 | 7 | 20.454 | 16.217 | 19.387 |
| 38 | 40 | 20 | 20 | 5 | 0.194 | 12 | 3 | 28.637 | 23.040 | 23.050 |
| 39 | 104 | 142 | 32 | 27 | 0.220 | 69 | 6 | 27.461 | 26.780 | 23.906 |
| 40 | 105 | 463 | 6 | 39 | 0.272 | 99 | 7 | 105.571 | 32.460 | 106.482 |
| 41 | 96 | 442 | 15 | 34 | 0.517 | 81 | 13 | 75.723 | 67.963 | 53.439 |
| 42 | 49 | 46 | 23 | 9 | 0.469 | 17 | 5 | 32.898 | 28.081 | 29.216 |
| 43 | 135 | 185 | 65 | 29 | 0.332 | 68 | 7 | 14.944 | 11.814 | 12.778 |
| 44 | 54 | 174 | 7 | 24 | 0.395 | 47 | 8 | 42.652 | 31.445 | 36.895 |
| 45 | 200 | 390 | 56 | 45 | 0.243 | 136 | 8 | 31.948 | 18.641 | 23.409 |
| 46 | 113 | 401 | 26 | 34 | 0.426 | 87 | 9 | 31.834 | 24.479 | 22.384 |
| 47 | 144 | 198 | 38 | 25 | 0.145 | 102 | 4 | 21.240 | 31.386 | 21.841 |
| 48 | 158 | 287 | 55 | 40 | 0.313 | 93 | 9 | 8.771 | 13.386 | 14.451 |
| 49 | 42 | 94 | 6 | 16 | 0.329 | 34 | 5 | 26.152 | 17.138 | 23.012 |
| 50 | 200 | 300 | 57 | 69 | 0.227 | 141 | 10 | 47.245 | 53.678 | 59.328 |

Set: protein sets 1-50; V: vertex number; E: edge number; Iso: number of isolated proteins; D_max_: maximal vertex degree; ClustC: cluster coefficient (global); Clust: maximal cluster size; Cliq: maximal clique degree; BP_max_: z_max_ for BP terms; CC_max_: z_max_ for CC terms; MF_max_: z_max_ for MF terms.


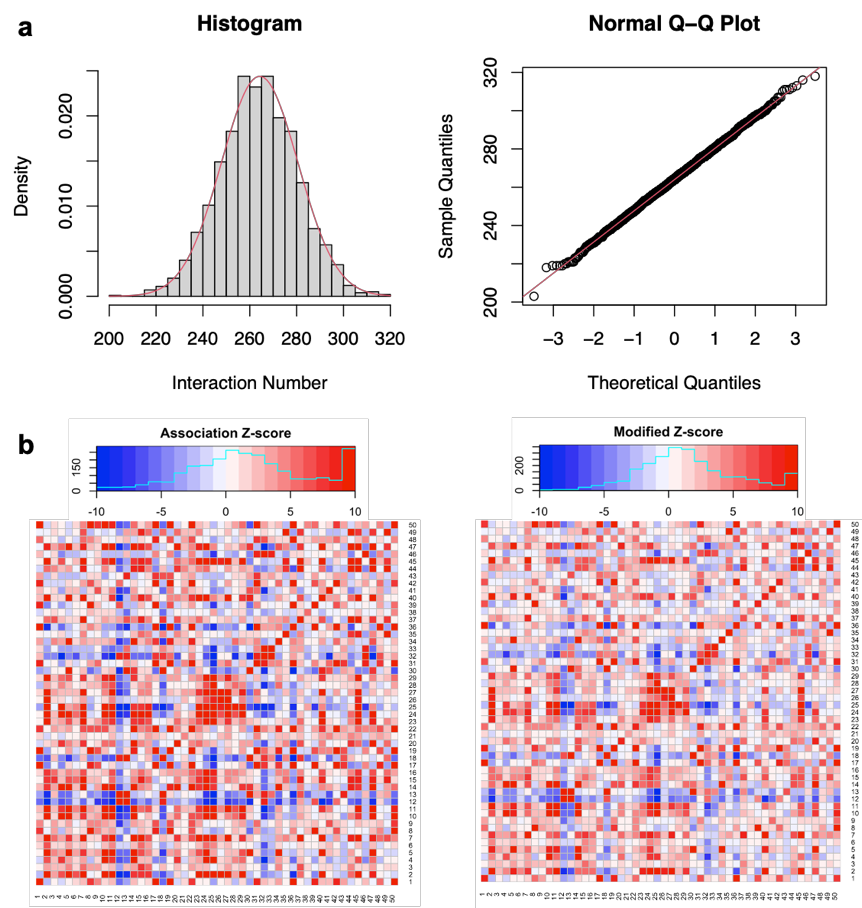


**Figure S1**. Normality test of the interaction numbers from null models and comparison of the Z-score and modified Z’-score heatmaps of the hallmark-hallmark interactions (a) Normal distribution of the interaction numbers between two randomly chosen hallmark gene sets in 2,000 null models. The Shapiro-Wilk normality test gives a p-value = 0.6778, accepting the null hypothesis of normal distribution. The left figure shows the histograms (grey) and fitted normal distribution (red) and the right figure shows the QQ-norm and QQ-line (red). (b) The original Z-score heatmap (left, also see Figure 2b) of the Hallmark-Hallmark associations versus the modified Z-score heatmap (right, i.e., Z’-score heatmap).


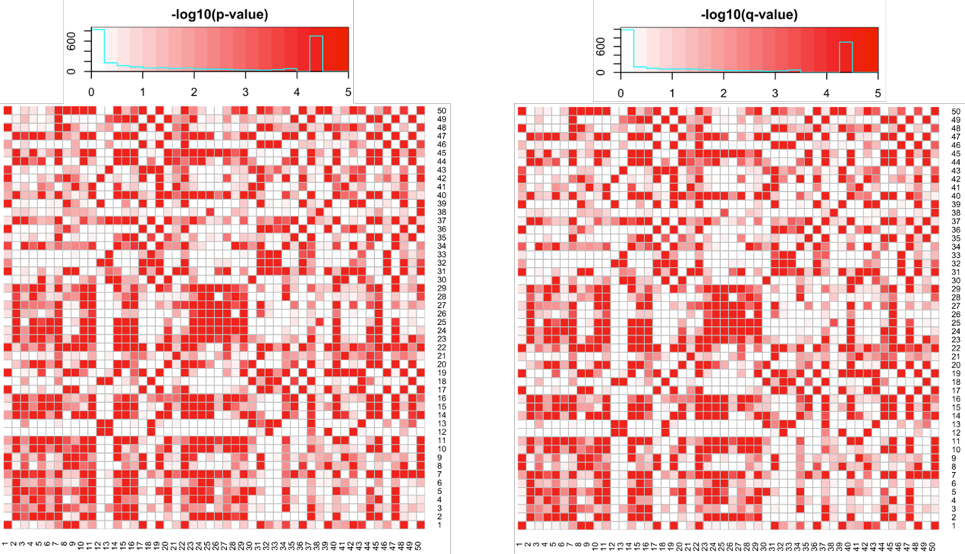


**Figure S2**. Heatmaps based on the p-values (left) and q-values (right) for evaluating the associations among the 50 hallmark gene sets. Both methods give qualitatively similar results.

**Figure S3**. Examples indicate that overrepresentation is not equivalent to enriched interactions, and that the interaction numbers from the PIN may be suppressed compared to the null network models. (a) Interactions between the hallmark sets 5 (blue) and 24 (red). In the PIN 344 interactions have been observed between these two sets; however, in the null models, these two sets have 204.5±13.5 interactions, leading to a Z=10.3 and p<1×10^-4^, despite both sets have no overlapping genes (J=0). (b) Interactions between the hallmark set 12 (blue) and 25 (red). These two sets have two overlapping genes (PDE4B and ADRM1, J=0.006). However, 117 interactions observed in the PIN and 366.3±18.4 interactions in the null models leading to a Z-score of -13.5 between these two sets.


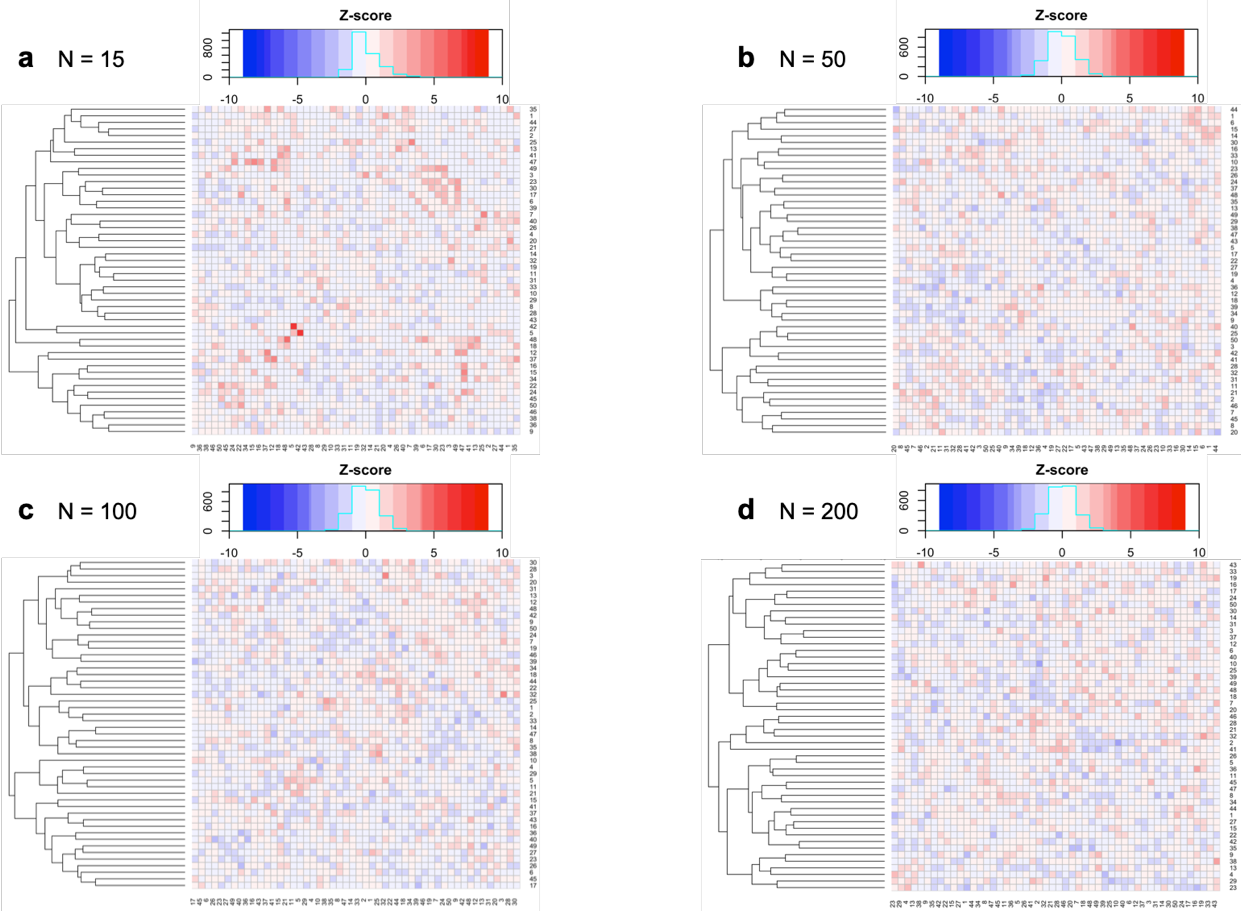


**Figure S4**. Interaction Z-score heatmaps of randomly constructed gene sets with (a) 15 (group I), (b) 50 (group II), (c) 100 (group III), and (d) 200 (group IV) genes for each set.


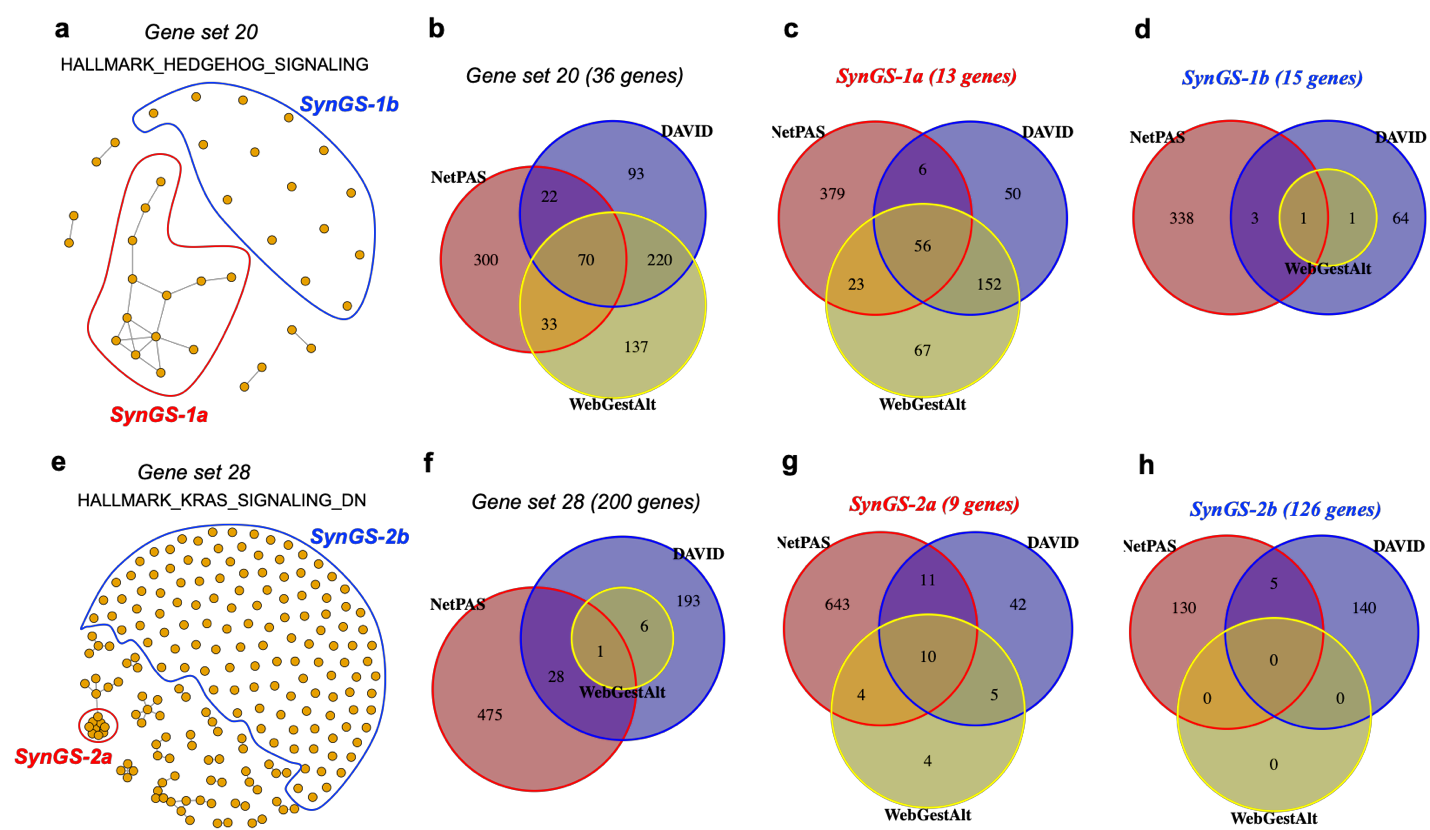


**Figure S5**. **Comparisons of NetPAS, DAVID and WebGestalt using synthetic gene sets.** (a) The synthetic gene set *SynGS-1a* (13 genes, circled in red) is the highly connected cluster, and *SynGS-1b* (15 genes, circled in blue) comprises all isolated genes taken from the hallmark set 20. (b) A 3-way Venn diagram of the enriched BP terms by NetPAS, DAVID and WebGestalt for hallmark set 20. (c) A 3-way Venn diagram by NetPAS, DAVID and WebGestalt for the *SynGS-1a* set, in which NetPAS showed more enriched BP terms than the hallmark set 20. (d) A 3-way Venn diagram by NetPAS, DAVID and WebGestalt for the *SynGS-1b* set. There is only one shared BP term, GO:0007399 ("nervous system development") shared by all three methods, and this term is shared by all three methods for both the hallmark set 20 and *SynGS-1a*. (e) The synthetic gene set *SynGS-2a* (9 genes, circled in red) is taken from the largest clique from the hallmark set 28, and the synthetic gene set *SynGS-2b* (126 genes, circled in blue) contains all isolated genes from the hallmark set 28. (f) A 3-way Venn diagram shows that for hallmark set 28, there are only 1 enriched BP terms, GO:0008015 (“blood circulation”) shared by the three methods NetPAS, DAVID and WebGestalt. (g) A 3-way Venn diagram shows that for the gene set *SynGS-2a* there are 10 enriched BP terms shared by NetPAS, DAVID and WebGestalt. However, the BP term GO:0008015 is only enriched by NetPAS (Z=14.049) but not by DAVID or WebGestalt. (h) For synthetic set *SynGS-2b*, none shared enrichment could be found among NetPAS, DAVID and WebGestalt.
